# Adrenocorticotropic hormone-dependent hypercortisolism caused by pancreatic neuroendocrine carcinoma: A thought-provoking but remorseful case of delayed diagnosis

**DOI:** 10.1016/j.ijscr.2021.105729

**Published:** 2021-03-05

**Authors:** Tomohide Hori, Katsutoshi Kuriyama, Hidekazu Yamamoto, Hideki Harada, Michihiro Yamamoto, Masahiro Yamada, Takefumi Yazawa, Ben Sasaki, Masaki Tani, Asahi Sato, Hikotaro Katsura, Yasuyuki Kamada, Ryotaro Tani, Ryuhei Aoyama, Yudai Sasaki, Yoko Iwasa, Masazumi Zaima

**Affiliations:** a Department of Surgery, Shiga General Hospital, Moriyama, 5-4-30 Moriyama, Moriyama, Shiga, 524-8524, Japan; b Department of Hepatobiliary Pancreatic Medicine, Shiga General Hospital, Moriyama, 5-4-30 Moriyama, Moriyama, Shiga, 524-8524, Japan; c Department of Diagnostic Pathology, Shiga General Hospital, Moriyama, 5-4-30 Moriyama, Moriyama, Shiga, 524-8524, Japan

**Keywords:** NEN, neuroendocrine neoplasm, ACTH, adrenocorticotropic hormone, Adrenocorticotropic hormone, Hypercortisolism, Neuroendocrine neoplasm, Pancreas, Carcinoma

## Abstract

**Introduction and importance:**

Definitive diagnosis of functioning neuroendocrine neoplasms (NENs) in the pancreas is challenging. Adrenocorticotropic hormone (ACTH) regulates adrenal cortisol production. Ectopic ACTH secretion by functioning NENs may cause hypercortisolism.

**Presentation of case:**

A 62-year-old woman who was receiving medications for hypertension and hyperlipidemia was referred to our hospital because of abnormal blood tests. Diabetes mellitus was initially diagnosed. Dynamic computed tomography and endoscopic ultrasound revealed a 35-mm diameter hypovascular tumor in the distal pancreas and multiple liver metastases. Endoscopic ultrasound-guided fine-needle aspiration resulted in a diagnosis of neuroendocrine carcinoma. The patient developed pancreatic leakage progressing to peritonitis, abscess formation, pleural effusion, and ascites after the fine-needle aspiration biopsy. Her clinical condition deteriorated to a septic state, necessitating emergency surgery comprising distal pancreatectomy, intraperitoneal lavage, and drainage. Wound healing was protracted and accompanied by ongoing high white blood cell counts and neutrophilia. She also developed a gastric ulcer postoperatively. Systematic endocrine investigations were performed because hypercortisolism caused by a functioning NEN was suspected. Eventually, a definitive diagnosis of an ACTH-producing NEN in the pancreas was made. Systemic chemotherapy was proposed; however, the patient and her family opted for palliative treatment only. She died 42 days after the initial diagnosis.

**Clinical discussion:**

We here present a patient with ACTH-dependent hypercortisolism attributable to a pancreatic NEN who died of progressive cancer after a delay in definitive diagnosis.

**Conclusion:**

Detailed investigation, including systematic endocrine examination and functional imaging studies, are important for precise diagnosis of, and appropriate treatment for, NENs.

## Highlights

• Diagnosis of functioning neuroendocrine neoplasms (NENs) in the pancreas is challenging.
• Adrenocorticotropic hormone (ACTH) regulates adrenal cortisol production.
• Functioning NENs may cause hypercortisolism as a result of ectopic ACTH secretion.
• Systematic endocrine examination and functional imaging studies are vital.
• Making a precise diagnosis enables appropriate treatment of NENs.

## 1 Introduction

Definitive diagnosis of functioning neuroendocrine neoplasms (NENs) in the pancreas is challenging [1,2]. However, precise diagnosis is crucial for selecting optimal treatment. Adrenocorticotropic hormone (ACTH) regulates adrenal steroid secretion, including cortisol production [3]. We herein report a patient with an ectopic ACTH syndrome caused by pancreatic neuroendocrine carcinoma, and discuss the diagnostic procedure that delayed arriving at a definitive diagnosis. This case was reported in accordance with the SCARE 2020 Guideline [4].

## 2 Presentation of case

**Fig. 1:**
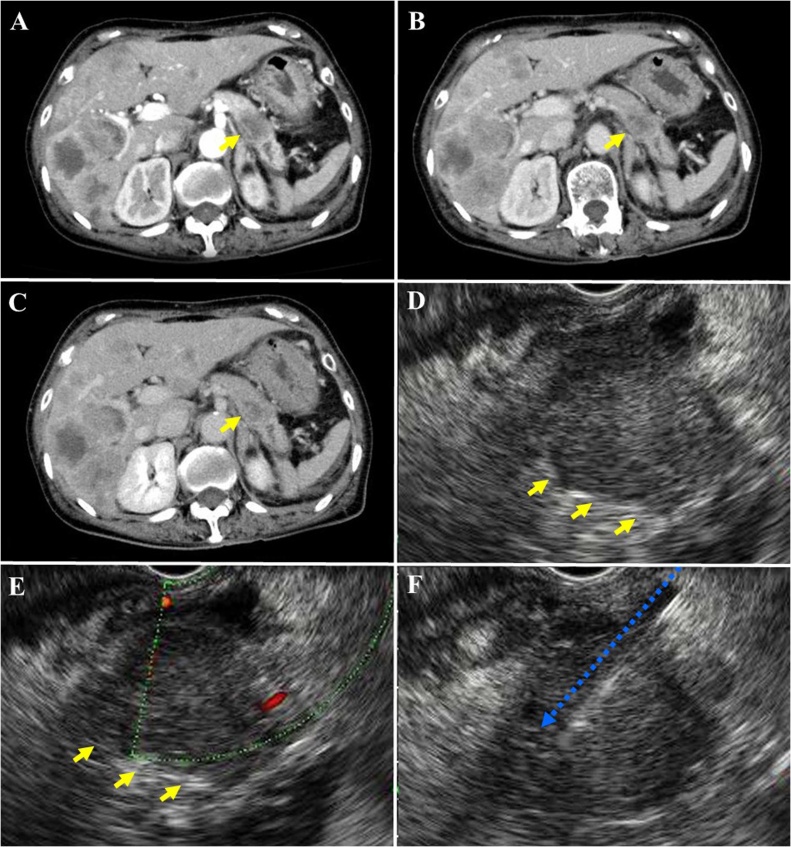
Findings of dynamic computed tomography and endoscopic ultrasound. Dynamic computed tomography revealed a hypovascular pancreatic tumor **(yellow arrows)** and multiple liver metastases **(A–C)**, as did endoscopic ultrasound **(E, F)**. Fine-needle aspiration was performed via the stomach **(F, blue arrows)**.

**Fig. 2:**
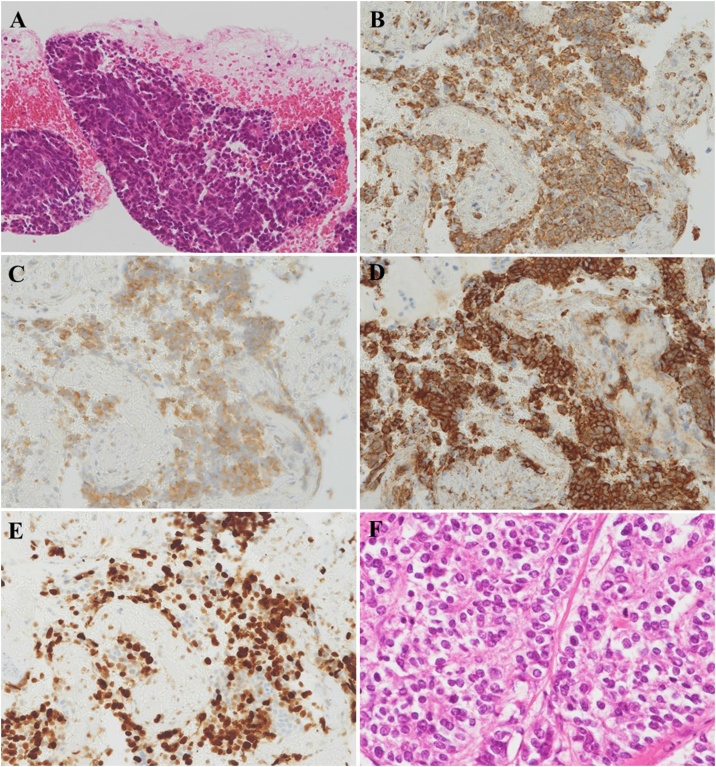
Histopathological and immunohistochemical findings. Photomicrographs showing cytological findings on hematoxylin- and eosin-stained sections **(A)** and immunohistochemistry staining for chromogranin A **(B,** ×40**)**, synaptophysin **(C,** ×40**)**, and CD56 **(D,** ×40**)**. The Ki-67 labeling index was >80 % **(E,** ×40**)**. Findings on pathological examination of the resected specimen (hematoxylin and eosin staining, ×40) were consistent with the preoperative diagnosis **(D)**.

A 62-year-old woman who was receiving medications for hypertension and hyperlipidemia was referred to our hospital because of abnormal blood tests (Table 1). Diabetes mellitus was diagnosed based on the results of blood examination. Dynamic computed tomography and endoscopic ultrasound revealed a 35-mm diameter hypovascular tumor in the distal pancreas and multiple liver metastases (Fig. 1). Endoscopic ultrasound-guided fine-needle aspiration was performed (Fig. 1). Cytological examination, including immunohistochemistry, revealed a tumor that was positive for chromogranin A, synaptophysin, and neural cell adhesion molecule 56 (CD56) (Fig. 2). The Ki-67 labeling index was >80 % (Fig. 2). A diagnosis of a T2N0M1 Stage IV neuroendocrine carcinoma was made in accordance with the tumor–node–metastasis classification [5]. The patient manifested pancreatic leakage progressing to peritonitis, abscess formation, pleural effusion, and ascites after the fine-needle aspiration, for which she received conservative treatment, including intravenous meropenem, for 7 days. However, her clinical condition deteriorated to a septic state, necessitating emergency surgery. Distal pancreatectomy was performed to remove the primary tumor. Additionally, infected necrotic tissue, including lymph nodes, was removed from the peripancreatic area. Intraoperative cytology revealed no malignant cells. Lavage of the peritoneal cavity was performed and drainage tubes placed intraperitoneally. Operative time was 176 min, and blood loss was 665 mL. Findings on histopathological and immunohistochemical examination of the resected specimen, including the Ki-67 labeling index, were consistent with a diagnosis of neuroendocrine carcinoma (Fig. 2). Wound healing was protracted. High white blood cell counts and neutrophilia persisted postoperatively, peak values being 24,000/μL and 23,086/μL, respectively. She also developed a gastric ulcer postoperatively.

**Table 1:** Results in blood examination.

		Normal range	Unit			Normal range	Unit
Aspartate aminotransferase	97	7−38	U/L	Carcinoembryonic antigen	44.2	<5	ng/mL
Alanine aminotransferase	115	4−43	U/L	Carbohydrate antigen 19−9	359.4	<37	U/mL
Lactate dehydrogenase	678	101−202	U/L	Growth hormone	0.53	0.13−9.88	ng/mL
Total bilirubin	1.44	0.22−1.20	mg/dL	Lutenizing hormone	0.14	0.09−0.38	IU/mL
γ-glutamyl transpeptidase	706	16−73	U/L	Follicle-stimulating hormone	0.26	0.25−1.13	IU/mL
Alkaline phosphatase	1001	103−335	U/L	Prolactin	35.50	3.12−29.3	ng/mL
Amylase	387	40−126	U/L	Cortisol	93.80	6.24−18.0	μg/mL
Lipase	465	13−49	IU/L	ACTH	292.0	7.2−63.3	pg/mL
Creatine phosphokinase	198	45−163	U/L	Antidiuretic hormone	8.6	<2.8	pg/mL
Albumin	3.3	3.9−4.9	g/dL	Estradiol	107.00	100−230	pg/mL
Creatinine	0.59	0.47−0.79	mg/dL	Antithyroglobulin antibody	<10	<28	IU/mL
Sodium	146	136−147	mmol/L	Anti-thyroid peroxidase antibody	<9	<16	IU/mL
Kalium	2.1	3.6−5.0	mmol/L	Dehydroepiandrosterone-sulfate	550	0.8−18.8	μg/mL
Total cholesterol	218	145−220	mg/dL	Somatomedin-C	28	129−304	ng/mL
Glucose	249	65−110	mg/dL	Gastrin	120	<200	pg/mL
Hemoglobin A1c	6.4	4.6−6.2	%	Glucagon	126	70−174	pg/mL
White blood cells	18600	3400−9200	/μL	Neuron-specific enolase	316.0	<16.3	ng/mL
Neutrophil leucocytes	17205	1800−7300	/μL	Thyroid-stimulating hormone	0.03	0.50−4.30	IU/mL
C-reactive protein	1.07	<0.05	mg/dL	Free thyroxine	0.67	0.70−1.70	ng/mL

**Abbreviation:** ACTH, Adrenocorticotropic hormon. The underlined values are all abnormal (out of normal range).

Detailed assessment of her endocrine status was performed because of the evidence of immunological dysfunction, protracted wound healing, diabetes mellitus, and development of a gastric ulcer. ACTH and cortisol concentrations were high (Table 1). Further immunohistochemical examination of the resected specimen revealed that the lesion was positive for ACTH, partially positive for somatostatin, and negative for insulin, glucagon, and gastrin. Hence, a definitive diagnosis of an ACTH-producing NEN in the pancreas was finally made. Systemic chemotherapy with long-acting somatostatin analogs or combination chemotherapy with 5-fluorouracil, leucovorin, irinotecan, and oxaliplatin was proposed. However, the patient and her family opted for palliative treatment only. She died of progressive cancer 42 days after the initial diagnosis.

## 3 Discussion

Adrenal steroid secretion is tightly regulated at multiple levels [3]. ACTH, which was first isolated in 1942 [6], is the primary regulator of cortisol production and is synthesized in the adrenal fasciculate cells [3]. Ectopic ACTH syndrome (also known as ectopic adrenocorticotropic syndrome) is responsible for approximately 10 %–20 % of cases of adult Cushing syndrome with hypercortisolism [7]. Hypercortisolism can cause morbid obesity, muscular weakness, compromised general condition, protracted wound healing, hypertension, impaired glucose tolerance or diabetes mellitus, hyperlipidemia, gastroduodenal ulceration, and osteoporosis [3].

Functioning NENs in the pancreas may produce hormones, including insulin, gastrin, vasoactive intestinal polypeptide, glucagon, somatostatin, serotonin and ACTH. Their clinical manifestations are largely determined by their hormone secretion profiles [8,9]. Despite our patient presenting with some symptoms consistent with hypercortisolism, it unfortunately took some time to recognize that she had hypercortisolism and then make the diagnosis of pancreatic NEN accompanied by ACTH-dependent hypercortisolism.

Multiple liver and extrahepatic metastases are already present at the time of initial diagnosis in >80 % of patients with pancreatic NENs [10]. Somatostatin receptor scintigraphy is useful for disease staging [11]. Our case was categorized as a Grade 3 neuroendocrine carcinoma according to the 2017 World Health Organization classification [12]. The prognosis of pancreatic NENs, including mixed neuroendocrine non-neuroendocrine neoplasms, is poorer than that of gastrointestinal and colorectal endocrine tumors [13]. Indeed, our patient with Stage IV pancreatic NEN actually had an extremely poor outcome.

Pathological or cytological assessment is crucial for precise diagnosis of pancreatic NEN [1,2]. Moreover, cytological assessment, including ascertaining the Ki-67 labeling index, would enable us to select an optimal treatment strategy [14]. In general, patients with advanced cancer have deteriorated immune system. Though we chose endoscopic ultrasound-guided fine-needle aspiration of the primary pancreatic neoplasm, metastatic tumors were also suitable target for liver needle biopsy in our case, and liver needle biopsy of metastatic tumor might be more safe in patient with deteriorated immune system.

Pancreatic leakage often accompanies with refractory symptoms, and subsequent intractable fistula may result in fatal outcome [15]. From the viewpoint of removal of leakage point of pancreatic juice with necrotic tissues, we chose distal pancreatectomy in this case. However, continuous lavage and peritoneal drainage might be better in this case. Although the treatment strategy was appropriate to make precise diagnosis, distal pancreatectomy was too invasive in advanced cancer patient with deteriorated immune system.

## 4 Conclusion

Making a definitive diagnosis of a functioning NEN in the pancreas is challenging. We herein review our patient’s clinical course and outcomes after infective complications of a biopsy procedure and subsequent delayed definitive diagnosis of ACTH-dependent hypercortisolism caused by a pancreatic NEN. We hope our case will provide a timely reminder for clinicians and surgeons.

## Declaration of Competing Interest

None of the authors have any financial conflicts of interest to declare.

## Funding sources

The authors declare that they received no funding support for this report.

## Ethical approval

Data were retrospectively evaluated. This report was approved by the Institutional Review Board of Shiga General Hospital, Moriyama, Japan.

## Consent

Written informed consent was obtained from the patient for publication of this case report and accompanying images. A copy of the written consent is available for review by the Editor-in-Chief of this journal on request.

## Author contribution

Tomohide Hori, PhD., MD., FACS. collected the data, and wrote the manuscript. All authors analyzed the data, and discussed therapeutic options, reviewed previous papers, and provided important opinions. T. Hori and M. Zaima supervised this report.

## Registration of research studies

Not Applicable.

## Guarantor

None.

## Provenance and peer review

Not commissioned, externally peer-reviewed.
